# Modeling the Ternary Complex TCR-Vβ/CollagenII(261–273)/HLA-DR4 Associated with Rheumatoid Arthritis

**DOI:** 10.1371/journal.pone.0011550

**Published:** 2010-07-14

**Authors:** Maria Cristina De Rosa, Bruno Giardina, Caterina Bianchi, Cristiana Carelli Alinovi, Davide Pirolli, Gianfranco Ferraccioli, Maria De Santis, Gabriele Di Sante, Francesco Ria

**Affiliations:** 1 Istituto di Chimica del Riconoscimento Molecolare, Consiglio Nazionale delle Ricerche, Rome, Italy; 2 Istituto di Biochimica e Biochimica Clinica, Università Cattolica del Sacro Cuore, Rome, Italy; 3 Dipartimento di Reumatologia, Università Cattolica del Sacro Cuore, Rome, Italy; 4 Istituto di Patologia Generale, Università Cattolica del Sacro Cuore, Rome, Italy; University of Wales Bangor, United Kingdom

## Abstract

**Background:**

It is known that genetic predisposition to rheumatoid arthritis (RA) is associated with the MHC class II allele HLA-DR4 and that residues 261–273 of type II collagen (huCollp261) represent an immunodominant T cell epitope restricted by the DR4 molecule. Despite recent advances in characterization of MHC and T cell receptor (TCR) contacts to this epitope, the atomic details of TCR/huCollp261/HLA-DR4 ternary complex are not known.

**Methodology/Principal Findings:**

Here we have used computational modeling to get insight into this interaction. A three-dimensional model of the TCR Vβ domain from a DR4^+^ patient affected by RA has been derived by homology modeling techniques. Subsequently, the structure of the TCR Vβ domain in complex with huCollp261/HLA-DR4 was obtained from a docking approach in conjunction with a filtering procedure based on biochemical information. The best complex from the docking experiments was then refined by 20 ns of molecular dynamics simulation in explicit water. The predicted model is consistent with available experimental data. Our results indicate that residues 97–101 of CDR3β are critical for recognition of huCollp261/HLA-DR4 by TCR. We also show that TCR contacts on p/MHC surface affect the conformation of the shared epitope expressed by DR alleles associated with RA susceptibility.

**Conclusions/Significance:**

This work presents a three-dimensional model for the ternary complex TCR-Vβ/collagenII(261–273)/HLA-DR4 associated with rheumatoid arthritis that can provide insights into the molecular mechanisms of self reactivity.

## Introduction

Recognition by T cell receptors (TCRs) of antigenic peptides (p) presented by class I or class II major histocompatibility complex (MHC) protein is central to cellular immune responses [1], [2]. The molecular events taking place at the TCR/p/MHC interface are also directly involved in immunomediated diseases. Rheumatoid arthritis (RA) is an autoimmune disease characterized by a chronic inflammation of the synovial joints leading to a progressive destruction of the articular cartilage that progressively invalidates patients [3]. Genetic predisposition to RA has been significantly associated with the HLA class II alleles HLA-DRB1 01 and HLA-DRB1 04 [4], [5]. DR β-chain encoded by these RA-related DRB1 genes possess a “shared epitope” formed by conserved amino acids at positions 67–74 [6]. It has been observed that sequence differences in this region, especially in residue 71, strongly influence T cell recognition and immune response [7], [8], [9] by determining the selection of peptides presented by the DR molecule [8].

Although the etiology of rheumatoid arthritis remains unknown, type II collagen is a strong candidate autoantigen. It is the predominant protein of articular cartilage, and autoimmunity to type II collagen is commonly detected in patients with RA [10], [11]. T cell responses in experimental collagen-dependent RA are directed towards the immunodominant pathogenic epitope encompassing residues 261–273 of collagen II [12], [13] (here-after called huCollp261).

Elucidating the mechanism underlying the molecular recognition of huCollp261/HLA-DR4 by TCR requires structural models of the complex. Till now, approximately 20 TCR/p/MHC complex structures have been solved and among these, eight are TCR/p/MHC of class II structures. Most contacts between TCR and peptide occur through the CDR3 loops, which exhibit the greatest degree of variability whereas the preponderance of generally conserved contacts with the MHC α helices is mediated through CDR1 and CDR2 [14]. So far, however, no crystal structure has been published for a TCR in complex with human type II collagen peptide/MHC assembly.

In our recent study, we used the VB-JB spectratyping (the so called “immunoscope” technique) to identify T cells specific for huCollp261 of DR4^+^ subjects in the early phases of RA [15]. This PCR-based technique divides a bulk T cell population in approximately 3000 groups on the basis of the VB and JB segments recombined and of the length of the CDR3 region that varies according to bases additions and subtractions at the V-D-J joint [16]. In the index DR4^+^ RA patient studied thoroughly by immunoscope [15], we identified and sequenced the CDR3 of a TCR β-chain belonging to T cells specifically stimulated by huCollp261 that are present in the blood and spontaneously enriched in the synovial fluid of inflamed joints. This TCR is obtained by recombination of VB1 and JB2.6 and has the following CDR3 region (VB1)CASS DTGS SGAN(BJ2.6). This sequence is termed here VB1^OE^. We also studied a DR4^−^DR1^+^ RA patient and found one T cell expanding specifically in response to huCollp261 that used a TCR obtained by recombination of the same VB1 and JB2.6, having a CDR3 region of the same length, but displaying a sequence (VB1)CASS GDRS AGAN(JB2.6). This sequence is termed here VB1^VB^. This TCR recognizes the same peptide but in a conformation different from the one recognized by VB1^OE^ since the DR molecule presenting it is certainly not DR4. The availability of two highly homologues sequences for TCR β-chains from DR4^+^ and DR4^−^ patients affected by RA gave us the opportunity to build a three-dimensional model of the ternary complex TCR-Vβ/huCollp261/HLA-DR4 by computational approaches. The VB1^VB^ sequence has been used as a sort of background sample that provides information about the non-specific contacts that can emerge when modeling the interaction of a VB1-JB2.6 TCR and the DR4/huCollp261 complex.

In the work presented here, reasonable structures of TCR Vβ domain and of huCollp261 bound to HLA-DR4 were first constructed by molecular modeling methods. Subsequently, we identified an equilibrated reasonable structure of the TCR-Vβ/huCollp261/HLA-DR4 ternary complex using protein-protein docking and molecular dynamics simulations. On the basis of the final complex structure and simulation results, we elucidated the molecular basis underlying the selectivity of the interaction within this ternary complex.

## Methods

### Ethics Statement

Informed written consent was obtained from all the patients. The research is in compliance with the Helsinki Declaration. The research was approved by the Institutional Ethical Committee of the Catholic University of the Sacred Heart.

### Patients and TCR Vβ domain sequencing

Patients OE and VB satisfied the American College of Rheumatology criteria for RA. Patients were characterized for the HLA-DR haplotype by PCR-SSO, using the Inno-LiPA HLA-DRB1 Amp Plus kit (Innogenetics N.V., 9052 Gent, Belgium), according to manufacturer's instructions.

In our previous work, we described the clinical characteristics of patient OE as well as TCR repertoire analysis and sequencing of the TCR Vβ domains of patient OE [15]. In this study we used the same protocols for TCR repertoire analysis and sequencing of the TCR Vβ domains of patient VB. DNA sequence was translated into protein sequence through the ExPASy Proteomics Server.

### Homology modeling of TCR Vβ domains

Sequences of TCR β-chains for which the three-dimensional structure was known were selected based upon similarity using PSI-BLAST (available on the World Wide Web at blast.ncbi.nlm.nih.gov/Blast). The homology model was based on the structure of human autoimmune TCR bound to a myelin basic protein self-peptide and a multiple sclerosis-associated HLA class II allele (Protein Data Bank (PDB) code: 1zgl) [17]. VB1^VB^ and VB1^OE^ were aligned with the crystallographic TCR Vβ domain of 1zgl using ClustalW (available on the World Wide Web at clustalw.genome.ad.jp/) and rendered using ESPript program [18]. Based on ClustalW alignments three-dimensional models of VB1^VB^ and VB1^OE^ were generated by comparative protein modeling with MODELLER [19] module in Discovery Studio Modeling1.1 (Accelrys Inc.).

Twenty models, optimized by a short simulated annealing refinement protocol available in MODELLER, were generated for each Vβ domain. The simulated annealing procedure was carried out *in vacuo* (dielectric constant  = 1) considering that in MODELLER, the effect of solvation, like electrostatics, is assumed to be encoded in the template structure and thus in the distance restraints derived from the template. The temperatures used in the simulated annealing procedure were 150 K, 250 K, 400 K, 1000 K for heating and 800 K, 600 K, 500 K, 400 K, 300 K for cooling.

The geometrical consistency of the model was evaluated based on PDF violations provided by MODELLER. The models were then evaluated using the programs VERIFY3D [20], PROCHECK [21] and by visual inspection using the computer graphics program Discovery Studio 2.1 (Accelrys Inc.)

### Molecular modeling of DR4/huCollp261 complex

Twenty conformations of huCollp261 (AGFKGEQGPKGEP, position 1, P1  =  F) bound to HLA-DR4 were generated using the simulated annealing protocol of MODELLER and HLA-DR4 in complex with human collagen peptide 1168–1180 (PDB code: 2seb) as starting structure [22]. The objective function used for structure generation in MODELLER is referred to as a molecular PDF (probability density function). This is a combination of all the feature PDFs used to restrain particular geometric features of the protein model. Molecular PDF values are collected to Table 1 and the best-ranked model based on PDF violations (Model 8) was selected.

**Table 1:** Molecular PDF and protein structure analysis for the twenty models of DR4/huCollp261.

		Ramachandran plot quality (%)			
Model	Molecular PDF	Core	Allowed	General	Disallowed
Model 8	2189.39	92.2	6.6	1.2	0.0
Model 11	2209.43	92.5	6.6	0.9	0.0
Model 12	2263.19	93.1	5.7	1.2	0.0
Model 15	2278.74	91.9	6.9	1.2	0.0
Model 10	2283.98	92.2	6.9	0.6	0.3
Model 4	2287.99	91.6	6.9	1.5	0.0
Model 16	2329.97	93.1	5.4	1.5	0.0
Model 20	2352.77	92.5	6.6	0.9	0.0
Model 9	2356.94	92.2	6.6	1.2	0.0
Model 3	2359.49	94.0	5.4	0.6	0.0
Model 5	2373.85	91.6	6.6	1.5	0.3
Model 13	2388.94	92.2	6.9	0.9	0.0
Model 2	2397.23	91.0	7.2	1.8	0.0
Model 19	2403.73	92.5	6.0	1.2	0.3
Model 7	2409.73	92.8	6.6	0.3	0.3
Model 18	2435.51	91.9	6.6	1.5	0.0
Model 17	2529.67	91.6	6.9	1.5	0.0
Model 14	2540.33	92.5	6.3	1.2	0.0
Model 6	3074.24	92.2	6.9	0.9	0.0
Model 1	3235.73	91.9	6.9	0.9	0.3

The quality of the structures was assessed using VERIFY3D [20] and PROCHECK [21] (Table 1).

The β105–β112 and β165–β168 sequences, which were not resolved in 2seb x-ray crystal structure, were built with an *ab initio* modeling routine of MODELLER [19].

### Protein-protein docking

Docking of all protein pairs was performed with the FTDOCK program [23]. FTDOCK is based on rigid-body geometric docking method originally proposed by Katchalski-Katzir et al. [24]. In this approach the relatively larger protein is held fixed while the smaller protein translates and rotates on a defined grid surface so as to establish the best geometric fit. FTDOCK uses fast Fourier transformation (FFT) grids to rapidly evaluate shape complementarity. A simple Coulombic model is then used as a binary filter, leaving only those complexes with attractive electrostatic interactions. Large positive FTDOCK scores denote complex formation with good surface complementarity, a score of zero indicates that the molecules do not interact at all and the score is negative if the smaller molecule significantly overlaps with the larger one. In all our calculations FTDOCK was run with electrostatics on using default parameters. For all protein pairs the rigid-body docking approach of FTDOCK resulted in 10,000 possible docked complexes which were reduced by a filtering procedure, based on biochemical knowledge. Interaction energies were computed using CHARMM [25] as implemented in DiscoveryStudio (Accelrys Inc.). The NACCESS program [26] was used to calculate the interface Accessible Surface Area (ASA).

### Validation of docking method

The methodology was first tested on the complex of the human TCR HA1.7 specific for the hemagglutinin antigen peptide (HA) from influenza A virus bound to class II molecule HLA-DR4 (PDB code: 1j8h) [27]. The system was digitized onto a 246×246×246 grid with the resolution of 0.69 Å. The distance constraint applied required the separation between the following residues and protein chains to be <4.5 Å: 30D:A 30D:B 50D:A 50D:B (97–98)D:P where A and B stand for α and β chain of HLA-DR4, respectively, D stands for β chain of TCR, P stands for HA peptide. This biological filter was selected based on the hypothesis of the two-step binding mechanism for T-cell receptor recognition of p/MHC complex for which the TCR binds p/MHC in a conserved diagonal orientation that first positions the CDR1 and the CDR2 loops mainly over the HLA-DR4 and then the CDR3 loops over the peptide [28]. The two-step binding mechanism is consistent with TCR/p/MHC crystal structures which show that the CDR1 and CDR2 loops primarily contact the MHC, whereas the highly diverse CDR3 loops mainly interact with the peptide [29].

The first part of the notation (30D:A 30D:B 50D:A 50D:B), therefore, which refers to the first step of the mechanism, means that we first isolated the diagonal orientations in which residues β30 and β50 of CDR1 and CDR2, respectively, are within 4.5 Å distance of any residue of MHC α and β chains. Subsequently, according to the second part of the notation ((97–98)D:P), related to the second step of the mechanism, we isolated the complexes for which residues β97 and β98, at the apex of CDR3, are closer than 4.5 Å to atoms of the antigen peptide.

Analysis of TCR/p/MHC crystal structures clearly indicates that residues β30 and β50 of CDR1 and CDR2, respectively, and β97 and β98 of CDR3 make the most frequent contacts for TCRs to p/MHC [29].

### Docking of TCR-VB1^VB^ and TCR-VB1^OE^ to huCollp261/HLA-DR4 complex

The systems were digitized onto a 206×206×206 (VB1^VB^) and 202×202×202 (VB1^OE^) grid with the resolution of 0.69 Å. The same filtering procedure of 1j8h complex was applied, which corresponded to: 32D:A 32D:B 52D:A 52D:B (100–101)D:P where A, B stand for α and β chain of HLA-DR4, respectively, D stands for β chain of TCR, P stands for huCollp261.

### Molecular dynamics simulations

The best structural model of the ternary complexes TCR-VB1^OE^/huCollp261/HLA-DR4 obtained from docking experiments, i.e. the model with the largest binding energy, largest interface ASA and that is likely to have biological meaning, was subjected to aqueous-phase MD simulations using GROMACS v.3.3.1 and employing the GROMOS96 force field [30]. The structure was immersed in a triclinic box with periodic boundary conditions and was solvated with explicit SPC water molecules. The system was then neutralized by 20 Na^+^ counterions that were added at random positions to the bulk solvent. The dimensions of the box (9.0 nm×9.3 nm ×11.4 nm) were set to allow at least 1.2 nm between protein and box faces on each side. The final system consisted of 5182 protein atoms surrounded by 30000 water molecules. Before running simulation, the system was energy minimized for 1000 iterations of steepest descents and then equilibrated for 20 ps, during which the protein atoms were restrained. All restraints were then removed from the complexes and the temperature of system was brought to 300 K in a stepwise manner: 10-ps long MD runs were carried out at 50, 100, 200 and 250 K before the production runs were started at 300 K. The total length of simulation was 20 ns. Berendsen coupling was employed to maintain a constant temperature of 300 K with a coupling constant τ of 0.1 ps. van der Waals interactions were modeled using 6–12 Lennard-Jones potentials with a 1.4 nm cutoff. Long-range electrostatic interactions were calculated using Particle Mesh Ewald method, with a cutoff for the real space term of 1.2 nm. Covalent bonds were constrained using LINCS algorithm. The time step employed was 2 fs and the coordinates were saved every 5 ps for analysis of MD trajectories which was carried out using the standard GROMACS tools g_rms, g_rmsf and g_hbond. In the use of g_hbond, a cutoff radius of 0.35 nm between donor and acceptor and a cutoff angle of 30° as geometric criteria were employed for the existence of a hydrogen bond.

This same MD protocol was applied to all VB1^OE^ models emerging from the docking filtering procedure in order to calculate the interface ASA at the end of the MD run.

### Computational alanine scanning

Residues important for the stabilization of the complex were identified using Baker's alanine scanning procedure [31], [32] and the Robetta web server (http://www.robetta.org). This approach calculates van der Waals' and electrostatic contributions to the free energy of binding. Positive values of ΔΔG means that the alanine mutation is predicted to destabilize the complex and negative values indicate a stabilizing effect. Binding energy “hot spots” are defined for residues at the subunit-subunit interface, whose Ala mutation causes a loss of free energy greater or equal to 1 kcal/mol.

## Results and Discussion

### Choice of TCR β-chains

The expansion of T cells specific for huCollp261 in DR4^+^ subjects was studied by “immunoscope”, in the peripheral blood mononuclear cells (PBMC), as previously reported for the only patient OE [15]. T cells carrying a rearrangement of VB1 and JB2.6 of 134b length were particularly interesting. This TCR is in fact enriched spontaneously in the inflamed synovia. In addition, its usage appeared specifically linked to DR4 haplotype and RA development. In fact, p139-specific cells using a rearrangement of this type were present in 4/6 DR4^+^ patients during acute presentation of the disease (3 samples) and remission (2 samples). Meanwhile, we did not find cells carrying this rearrangement in any of 5 DR4^+^ healthy donors.

In a separate set of experiments, not previously reported, we tested 4 more subjects, 2 DR1^+^ DR4^−^ RA patients, 1 DR1^−^ DR4^−^ RA patient and 1 patient suffering from acute arthritis of other origin. Out of this latter group of patients, one DR4^−^DR1^+^ RA patient (patient VB) displayed the usage of a VB1-JB2.6 of 134b length in response to stimulation with huCollp261. Since T cells from this patient recognized huCollp261 most likely in the contest of DR1, we sequenced and modeled the VB1-JB2.6 134b TCR β-chain from this patient to obtain a control for the specificity of the modeled interaction between the TCR β-chain obtained from DR4^+^ patient OE, huCollp261 and the DR4 molecule itself.

### Modeling of TCR Vβ domains and of huCollp261/HLA-DR4 complex

**Figure 1:**
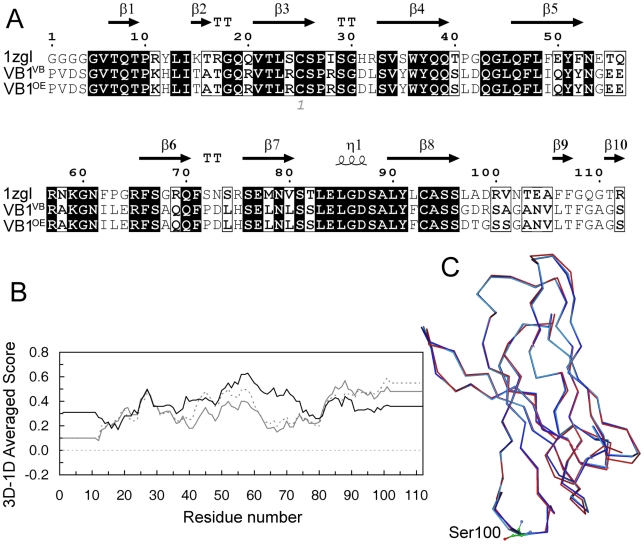
Modeling of TCR-VB1^VB^ and TCR-VB1^OE^ domains. A) Sequence alignment of VB1^VB^ and VB1^OE^ with 1zgl Vβ domain. Alignments were performed with ClustalW algorithm and ESPript software. Identical and similar amino acids are in *dark* and *white* boxes, respectively. The secondary structural elements are shown aligned to their respective sequences. Numbering of amino acids begins with the first amino acid residue of sequenced VB1^VB^ and VB1^OE^; B) Residue-based quality assessment results obtained by the Verify3D program using the coordinates of 1zgl crystal structure (*black*) and the coordinates of the three-dimensional models of VB1^VB^ and VB1^OE^ (*continuous grey* and *dashed grey,* respectively). *X axis*: amino acid numbering of VB1^VB^ and VB1^OE^ starting from the first sequenced residue. *Y axis*: average three/one-dimensional scores for residues in a 21-residue sliding window; C) Cα trace of VB1^VB^ (*dark blue*) and VB1^OE^ (*light blue*) model structures superimposed onto the Cα trace of template 1zgl Vβ domain (*red*). Ser100, at the apex of CDR3 loop of VB1^VB^ and VB1^OE^, is shown in ball and stick representation.

Homology models of VB1^VB^ and VB1^OE^ domains were constructed with the program MODELLER which implements an automated approach to comparative protein structure modeling by satisfaction of spatial restraints. The program automatically generates a set of restraints that includes the CHARMM forcefield, statistical preferences mined from PDB, and distance and dihedral restraints extracted from aligned templates, than generates a set of models which are consistent with all restraints. To find template structures, a specific BLAST sequence search of the Protein Data Bank (PDB) using default parameters was performed. The convergence matrix used to search the PDB produced significant alignments with the autoimmune TCR bound to a myelin basic protein self-peptide and a multiple sclerosis-associated HLA class II molecule (PDB code: 1zgl; identity 62%, similarity 75% with VB1^VB^; identity 63%, similarity 76% with VB1^OE^), the TCR Vβ5.1 in complex with staphylococcal enterotoxin K (SEK) (PDB code: 2nts; identity 62%, similarity 75% with VB1^VB^; identity 63%, similarity 76% with VB1^OE^), the TCR in complex with an anti-TCR Fab fragment derived from a mitogenic antibody (PDB code: 1nfd; identity 58%, similarity 72% with VB1^VB^; identity 58%, similarity 72% with VB1^OE^). The 1zgl complex in which human TCR is bound to HLA-DR2 protein appeared the appropriate template for comparative modeling of VB1^VB^ and VB1^OE^ β chains. The alignment of VB1^VB^ and VB1^OE^ domains with 1zgl generated by ClustalW is reported in Fig. 1A. The twenty different models generated for each Vβ domain by MODELLER were carefully analyzed for energy value and probability density function (PDF) violations. Among the first three original models, which were quite similar with respect to violations and energy values, the model possessing the least root mean square deviation (RMSD) value with the backbone atoms of 1zgl (0.23 Å for VB1^VB^ and 0.26 Å for VB1^OE^), was selected for further study. The goodness of the folding was assessed by VERIFY-3D, which evaluates the compatibility of a given residue in a certain three-dimensional environment. As shown in Fig. 1B, the three/one-dimensional scores of our models are always positive and are similar to those obtained with the template structure of 1zgl. PROCHECK analysis indicates that the quality of the Ramachandran plots (97.9% of the residues in the allowed regions) were equivalent to those of the template structure. The superimposition of the modeled TCR Vβ structures is reported in Fig. 1C. CDR3 Ser100 of both VB1^VB^ and VB1^OE^ is positioned at the apex and appears most accessible for interaction with peptide/HLA-DR4 complex. The flanking residues (Arg99 and Ala101 in VB1^VB^, Gly99 and Ser101 in VB1^OE^) may play important roles in the packing of the CDR3 loop structures.

**Figure 2:**
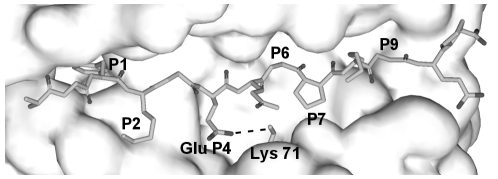
Model of huCollp261 peptide in the HLA-DR4 binding cleft. The computationally generated model closely resembles the hypothetical model suggested by Dessen et al. (22) for the DR4 recognition of huCollp261. Phe263 fits into the P1 pocket and Glu266 (P4) hydrogen bonds to Lys71β. The MHC peptide-binding groove is represented with Connolly solid surface, whereas the ligand peptide is shown in stick representation.

Modeling of huCollp261 peptide (AGFKGEQGPKGEP, position 1, P1  =  F) bound to HLA-DR4 was performed using the simulated annealing protocol of MODELLER and the crystallographic complex between human collagen peptide (CII) 1168–1180 and HLA-DR4 as starting structure. It is worth noticing that the automatically generated three-dimensional model (Fig. 2) displays the same structural features proposed for the DR4 recognition of huCollp61 by Dessen et al. [22] simply on the basis of the analysis of the MHC class II structures determined to date. In fact, observing that the peptides in complexes with human and murine MHC class II molecules have remarkably similar conformations, with P1, P4, P6, P7 and P9 partially buried in pockets and P-2, P-1, P2, P3, P5, P8, P10 and P11 substantially exposed to solvents, Dessen et al. hypothesize how the CII(261–273) peptide could be aligned and modeled from the DR4/CII(1168–1180) structure. In agreement with Dessen et al. working hypothesis, in our computationally-generated model Phe263 (P1) of huCollp261 is bound in the large nonpolar pocket 1 which was occupied by Met in the CII(1168–1180)/DR4 complex; Glu266 (P4) is hydrogen bonded to Lys71β as does P4 Asp in CII(1168–1180); Pro269 (P7) fits in the shallow pocket 7 (Fig. 2). As expected, Gln267 (P5) and Lys270 (P8), extend prominently into solvent where can be contacted by TCR (Fig. 2). In addition, as hypothesized [22], CII(1168–1180) main-chain hydrogen bonds to DR4 are maintained in the model.

### Docking validation

To validate the docking method, we first applied our technique to the rebuilding of a known TCR/p/MHCII crystallographic structure. The complex of TCR Vβ domain specific for the hemagglutinin antigen peptide (HA) with HLA-DR4 (PDB code: 1j8h) was selected for this purpose. FTDOCK was used to scan the relative orientations of the molecular complex in a systematic way, and produced 10,000 docking orientations. For the 10,000 docked models, we calculated the root mean square deviation (RMSD) of Cα atoms of each model structure from the native complex structure (1j8h). We observed that there are only two complexes with RMSD <4.5 Å (2.43 Å and 4.27 Å) and 17 complexes with 4.5 Å < RMSD <8 Å. The rest have very high RMSD.

The 10,000 structures were then filtered by the distance constraints using available biological information. We first took into account the the two-step binding mechanism [28] for TCR recognition as a means of filtering the docking results. In this model, initial TCR-MHC interactions, aided by minor contributions from TCR contacts with the peptide, guide the TCR to its ligand. This is followed by a final folding of the CDR3 loops of the TCR over the peptide [28]. Analysis of TCR/p/MHC crystallographic structures [29], which is consistent with the two-step binding mechanism, indicated the constraints for filtering procedure. We initially eliminated the models in which residues Asnβ30 and Aspβ50 of CDR1 and CDR2, respectively, were positioned away (>4.5 Å) from MHC α and β chains; then, among the remaining predictions, only those that had a distance less than 4.5 Å between Leuβ97 and Proβ98 of CDR3 and any residue of the HA peptide, were kept for further analyses. The eight remaining models were examined using the molecular graphics display program DiscoveryStudio (Accelrys Inc.) and a further biological filter was applied following the structural analysis of Deng and Mariuzza [33] which points out how the three CDR loops of Vβ contact the central and C-terminal portion of peptide. Therefore, complexes for which the CDR3β does not focus on the central portion of the MHC-bound peptide (P5-P6) were eliminated [33]. This left four models which were energy-minimized. Among these, the complex possessing the largest interaction energy between TCR Vβ and p/MHC and the greatest buried surface area is the same model structure exhibiting the lowest RMSD among the original 10,000 docked models (Cα RMSD  = 2.43 Å). We were thus confident that FTDOCK can generate TCR/p/MHC model complexes close to native structures and this procedure was chosen to study the interaction of VB1^VB^ and VB1^OE^ TCR domains with huCollp261/HLA-DR4 complex.

### Docking of VB1^VB^ and VB1^OE^ domains with huCollp261/HLA-DR4 complex

**Figure 3:**
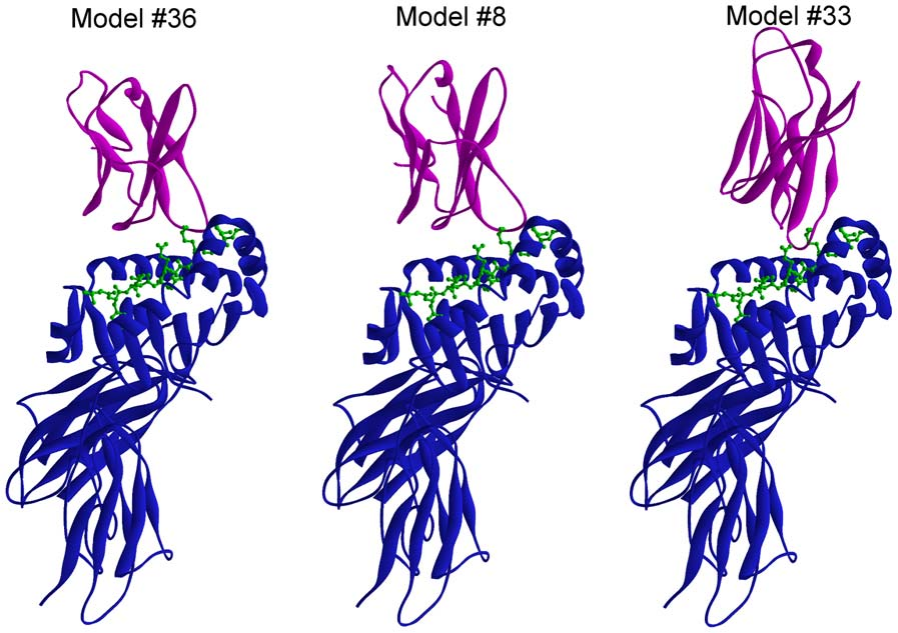
Molecular docking results. Structural comparison of overall structures of TCR-Vβ/huCollp261/HLA-DR4 complex models #36 (VB1^VB^), #8 (VB1^OE^) and #33 (VB1^OE^) emerging from the docking study. Color coding is as follows: *magenta*, TCR-Vβ domain; *blue*, HLA-DR4; *green*, peptide.

The same docking protocol and filtering procedure successfully used for method validation was then employed to investigate the docking of VB1^VB^ and VB1^OE^ domains with huCollp261/HLA-DR4 complex. The selection of residues for filtering constraints based on the two-step binding mechanism for TCR recognition [28] and on the analysis of TCR/p/MHC crystal structures [29]. Therefore, to narrow down the predicted 10,000 conformations of FTDOCK to a manageable number we first requested Leuβ32 and Tyrβ52 (corresponding to TCR HA1.7 Asnβ30 and Aspβ50, respectively) to be positioned at a distance less than 4.5 Å from MHC α and β chains. Then, according to the second step of the mechanism, we included only orientations where the peptide was within 4.5 Å distance from VB1^VB^ Ser100 and Ala101 and from VB1^OE^ Ser100 and Ser101 (corresponding to TCR HA1.7 Leuβ97 and Proβ98, respectively). In doing so, seven complexes remained for both VB1^VB^ and VB1^OE^. Interaction energies and interface Accessible Surface Area (ASA) for the selected minimized complexes are reported in Table 2. In the case of VB1^VB^, only three candidate complexes (namely models #17, #21 and #36 in FTDOCK ranking) show the Vβ domain located over the central and C-terminal portions of the peptide. Analogously, of the seven candidate conformations for VB1^OE^ complex, only three (namely model #8, model #33 and model #37 in FTDOCK ranking) display the Vβ domain poised above the C-terminal half of the peptide. Considering that a higher interface ASA is an indication of a higher shape complementary [26] between the molecules, the models with the largest binding energy and interface ASA were chosen as the most reasonable orientation. From the analysis of Table 2, model #36 in FTDOCK ranking was the most reasonable model for VB1^VB^ and model #33 was the most reasonable for VB1^OE^. Visual inspection of candidate conformations reported in Table 2 also showed that the binding conformations #36 (VB1^VB^) and #8 (VB1^OE^) are very similar with overall RMSD of 1.36 Å when the Cα atoms are optimally aligned. The three models emerging from the docking studies, namely #36^VB^, #8^OE^ and #33^OE^ are reported in Figure 3. If we look at the regions of the proteins involved in the interaction, we see an important difference among the models. CDR3β of model #36^VB^ and #8^OE^ focus on the peptide C-terminus, whereas in model #33^OE^ CDR3β is positioned above P5-P6 of peptide as observed in binding topologies of autoimmune complexes [33]. As stated in the introduction, VB1^VB^ recognizes huCollp261 bound to an HLA molecule different from DR4. Model #36^VB^, therefore, provides a sort of background model of the non-specific interaction that any TCR β-chain, generated by recombination of VB1 and BJ2.6 of the same length, engages with the huCollp261/HLA-DR4 complex. Since model #8^OE^ is very similar to this non-specific interaction, we selected model #33^OE^ as the complex structure for TCR-VB1^OE^/huCollp261/HLA-DR4 complex most likely provided of biological meaning.

**Table 2:** Interaction energy and interface Accessible Surface Area (ASA) in the minimized complexes emerging from docking calculations.

BV1^VB^			BV1^OE^		
FTDOCK score	Eint (kcal/mol)	ASA (Å^2^)	FTDOCK score	Eint (kcal/mol)	ASA (Å^2^)
8	−46.6	1176.5	8	−45.3	1181.1
17	−53.8	1224.6	13	−46.7	851.4
21	−56.6	1298.6	14	−54.5	1247.9
33	−34.6	1201.3	29	−43.5	977.0
36	−62.5	1370.5	33	−63.7	1384.8
52	−41.5	1065.3	34	−56.0	1198.1
56	−50.4	1235.2	37	−54.4	1077.0

### Molecular dynamics simulation

**Figure 4:**
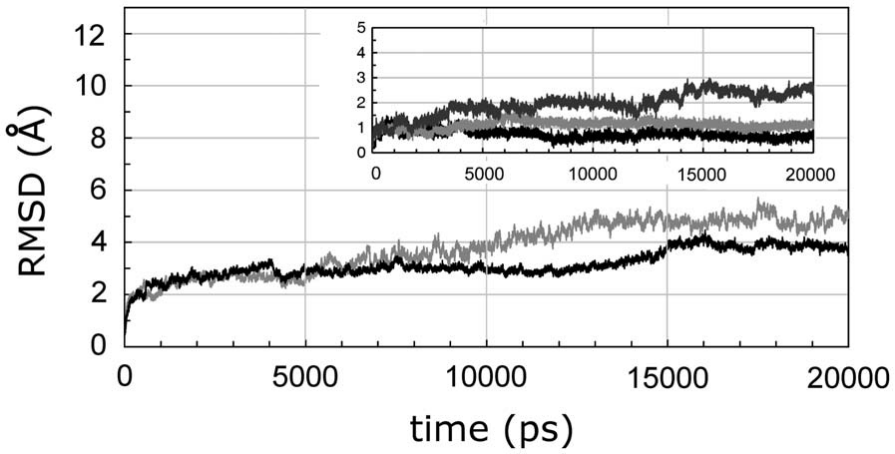
Cα- RMSDs versus time. The time evolution of the RMSD values for huCollp261/HLA-DR4 complex (*grey line*) and VB1^OE^ TCR (*black line*). *Inset* shows individuals CDR loops: CDR1 (*black line*), CDR2 (*light grey*) and CDR3 (*dark grey*) computed through the 20 ns MD simulation of VB1^OE^ TCR/huCollp261/HLA-DR4 ternary complex.

The model emerging from the docking was further investigated by MD simulation. Evaluation of structural drift is provided by analysis of the Cα atom RMSDs from the initial structures as a function of time. The RMSDs of the VB1^OE^ TCR and of the huCollp261/HLA-DR4 complex through the 20 ns trajectory were computed with respect to their corresponding initial structures. The Cα RMSD values fluctuated for the last 5 ns of the MD run around values of 3.9±0.1 and 4.8±0.2 Å for VB1^OE^ TCR and huCollp261/HLA-DR4, respectively (Fig. 4). The moderately large RMSD of the bound-MHC and, to a lesser extent, of the bound TCR Vβ domain indicates the existence of conformations that are somewhat different from the starting structure. A closer inspection of the RMSD for the HLA-DR4 molecule indicates that its variation is mainly due to the contribution of the several loops in the β2 domain. Analysis of RMSD for the TCR's CDRs (Fig. 4, *inset*) indicates that CDR1 and CDR2, which are positioned almost exclusively over the DR4 helix α1, show high stability reaching a plateau of 0.7 Å and 1.1 Å already after 5 ns. The CDR3 shows a higher deviation (Cα RMSD values fluctuating around values of 2.4±0.2 Å in the last 5 ns) indicating that it significantly contributes to the overall deviation of the TCR β-chain. The RMSDs of the CDRs correspond to a structural feature of TCR/p/MHC complexes in which the CDR1 and CDR2 loops are significantly more rigid than CDR3 loop and show little or no rearrangement upon binding to the p/MHC complex. In contrast, the CDR3 loop appears highly flexible and mobile and undergoes substantial repositioning upon binding the p/MHC complex. Such a behavior of CDRs is consistent with the reported observation that CDR loops of the TCR display different conformations in the free and bound states [34].

### Interactions at the protein surface

The average structure over the last 5 ns of simulation was used to analyze the interactions at the protein surface. The interface ASA value resulted significantly increased (1686.5 Å^2^) indicating that the molecular dynamics run induced a significant improvement in the surface matching. All the VB1^OE^ models emerging from the docking (Table 2) were further investigated by MD simulations to the aim of better evaluating the enhancement of interface ASA in the selected model #33^OE^. The interaction energy and interface ASA for the seven VB1^OE^/huCollp261/HLA-DR4 complexes averaged over the MD simulation runs are reported in Table 3. By comparing Tables 2 and 3, we realize that the ASA value resulted essentially increased in models #8, #13, #29, #33 and #34, while a less pronounced increase in the ASA is observed for model #14; a decrease being instead registered for model #37. Notably, the selected model #33 proved to be the model with the largest binding energy and interface ASA also at the end of the MD simulations, thus confirming the validity of the model selection.

**Table 3:** Interaction energy and interface Accessible Surface Area (ASA) for the VB1^OE^/huCollp261/HLA-DR4 complexes averaged over the MD simulation runs.

FTDOCK score	Eint (kcal/mol)	ASA (Å^2^)
8	−114.1	1645.4
13	−67.4	1104.9
14	−108.7	1340.2
29	−90.3	1517.2
33	−126.8	1686.5
34	−93.4	1483.6
37	−66.4	964.5

Interactions between TCR and huCollp261/DR4 complex are mainly restricted to van der Waals contacts, with limited juxtaposition of hydrophobic surfaces (Table 4). Of note, several TCR Vβ residues make contacts to HLA-DR4 Gln57α, Ala61α and Gln70β, which stand out as MHC class II conserved contact residues from the analysis of TCR/p/MHC crystallographic complexes [29].

**Table 4:** van der Waals contacts† between VB1^OE^ and huCollp261/HLA-DR4.

VB1^OE^	HLA-DR4	huCollp261
Ser29	Ala61α, Ala64α, Val65α, Ala68α	Pro269
Gly30	Ala61α, Val65α	
Leu32	Gln57α, Ala61α	
Tyr52	Gln57α, Leu60α	
Arg57	Gln57α	
Thr98	Gln57α, Gly58α, Ala61α	
Gly99		Gln267
Ser100	Gln70β	Gln267, Gly268, Pro269
Ser101	Leu67β, Gln70β, Lys71β	Glu266, Gln267, Gly268, Pro269
Gly102	Leu67β, Gln70β	Pro273
Ala103	Gln70β	Pro269, Pro273
Asn104	Asp66β	

† van der Waals contacts are ≤4.0 Å.

Identity of residues 67–74 of MHCII β-chain in DR4 and DR1 (“shared epitope” region) has been correlated with increased risk for RA [35]. In the predicted model we observe that some residues of the CDR3 loop 97–101 of VB1^OE^ (DTGSS) form van der Waals contacts with this “shared epitope” region. As found for recognition of a myelin basic protein self-peptide by TCR 3A6 (PDB code: 1zgl) [17], few hydrogen bonds are observed between TCR Vβ domain and the huCollp261 peptide (Table 5), a feature that likely contributes to low affinity binding and the observed crossreactivity in autoimmune TCRs [36], [37].

**Table 5:** Intermolecular hydrogen bonds between VB1^OE^ and huCollp261/HLA-DR4.

VB1^OE^	huCollp261	HLA-DR4	Frequency (%)†
Tyr52 (OH)		Gln57α (NE2)	52.24
Arg57 (NH2)		Gln57α (NE2)	17.22
Arg57 (NH1)		Gln57α (NE2)	20.74
Ser100 (O)		Gln70β (NE2)	52.24
Ser101 (OG)		Gln70β (O)	17.22
Ser101 (OG)	Glu266 (OE1)		20.74
Ser101 (N)	Gln267 (O)		52.36
Gly102 (N)		Gln70β (OE1)	81.62

† Interactions were statistically monitored throughout the MD trajectory for a total of 5000 conformations.

**Figure 5:**
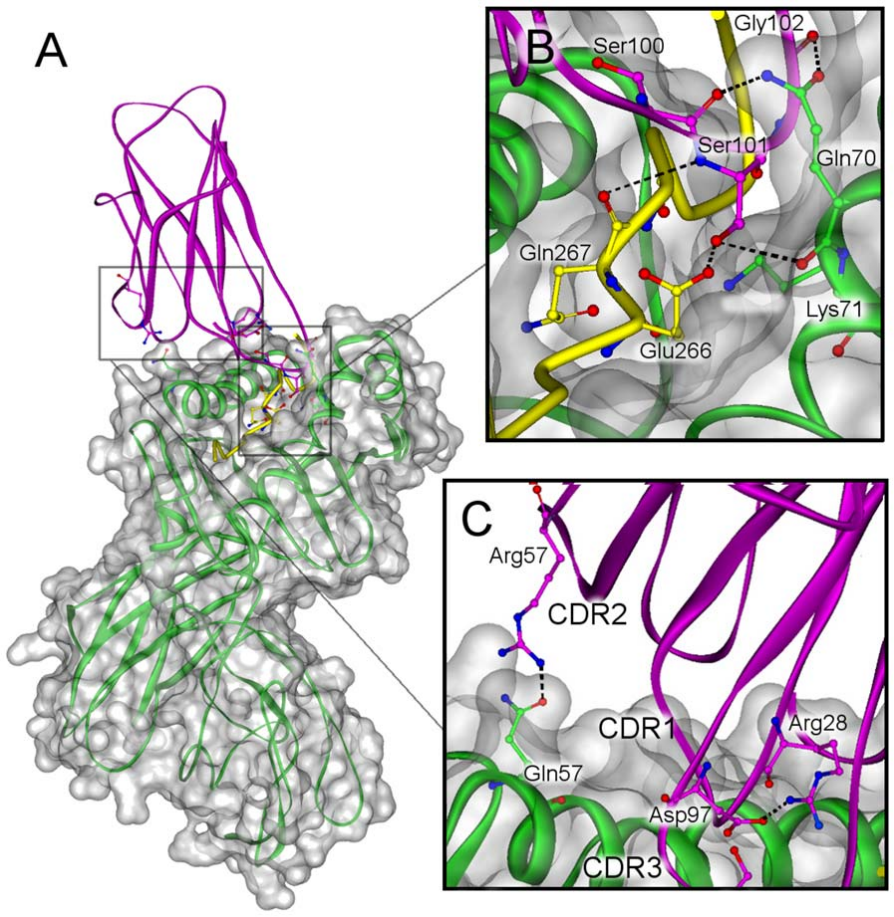
Positioning of the TCR VB1^OE^ domain over the huCollp261/HLA-DR4 complex. A) Overall three-dimensional structure of TCR-VB1^OE^/huCollp261/HLA-DR4 ternary complex generated using molecular dynamics simulations. The backbone structure of VB1^OE^ (*magenta*) and HLA-DR4 (*green*) are displayed in solid ribbon representation; huCollp261 (*yellow*) is shown in tube representation. HLA-DR4 is represented with Connolly transparent solid surface; (B) Zoom view of the peptide binding cleft showing the hydrogen-bonding interactions involving CDR3β; (C) Zoom view of the binding surface showing TCRβ residues important for the stabilization of the complex. Atoms are shown in ball and stick representation and colored by atom type with the exception of C atoms, colored by subunit.

Our MD simulation showed that peptide contacts are made primarily through the CDR3 loop (Table 4, Table 5) which overlays the central region of the peptide-binding groove (Fig. 5A). The side chain of peptide Glu266 (P4) is the first CDR3-contact residue and forms a hydrogen bond with Ser101 OG atom (Fig. 5B). At various points during the molecular dynamics run, Ser101 OG atom alternately forms hydrogen bonds with Glu266 OE1 atom and the carbonyl oxygen atom of DR4 Gln70β (Fig. 5B). Due to the binding of TCR Ser101β to Glu266, Lys71β of MHC “shared epitope” moves away from Glu266 causing a separation of 8.8 Å between the side chains of Lys71 and Glu266, a distance that appears to disfavor a direct contact. Thus, the Lys71β-Glu266 interaction, suggested by Dessen [22] and by our modeling (Fig. 2) is significantly affected by TCR binding. The second CDR3-contact residue of huCollp261 is Gln267 (P5) whose backbone oxygen atom is hydrogen bonded to Ser101 N atom (Fig. 5B). Gly268 (P6), Pro269 (P7) and Pro273 (P11) provide much weaker interactions to the modeled TCR, with Pro269 and Pro273 loosely interacting with the hydrophobic Ala103 (Table 4).

**Figure 6:**
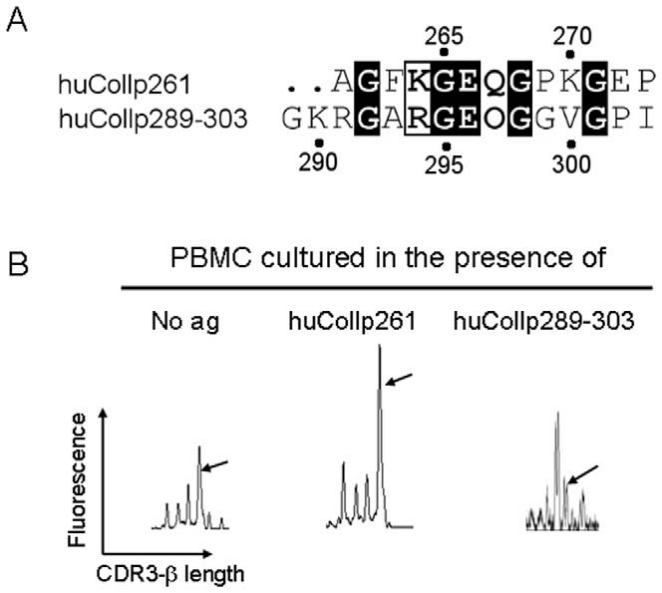
T cells carrying the BV1-BJ2.6 TCR β chain do not expand in response to stimulation with peptide huCollp289–303. A) Sequence alignment of peptide huCollp261 with huCollp289–303 performed with ClustalW algorithm and ESPript software. Identical and similar amino acids are in *dark* and *white* boxes, respectively. B) BV1-BJ2.6 spectra obtained from peripheral blood mononuclear cells (PBMC) of patient OE stimulated *in vitro* in the absence of added antigen (background) or in the presence of 20 µg/ml of peptides huCollp261 or huCollp289–303. PBMC were obtained from patient OE during a clinical relapse of disease. They were cultured and cDNA was obtained. BV-BJ spectratyping for rearrangements of BV1 and BJ2.6 was performed as described [15]. The spectra report the distribution of each TCR βCDR3 as a function of its length, where peaks are separated by a 3-base, i.e. one amino acid, difference. The fluorescence of each peak is a function of the amount of CDR3 of each length. Arrows indicate the 134b peak corresponding to the TCR β-chain under study.

We had the opportunity to test the hypothesis that Gln267 may play a relevant role in the recognition of huCollp261 by the TCR under study, by stimulating in parallel PBMC from patient OE with a peptide encompassing a subdominant epitope of the same protein, namely peptide huCollp289–303 (sequence GKRGARGEOGGVGPI, where O is hydroxyproline, Hyp). We showed that 25% of huCollp261-specific T cells recognize also an epitope contained in peptide huCollp289–303 [15]. In Figure 6, panel A, the sequence alignment between huCollp261 and huCollp289–303 is shown. A functional study [38] identified the “core” epitope recognized by T cells within peptide 259–273 of human collagen II in the region encompassing residues 263–268. We can observe that there are three amino acid residues that are conserved between huCollp261 and huCollp289–303 within this area, and that Phe263 of huCollp261 (the most functionally relevant residue for binding to HLA-DR4 according to the same study [38]) is aligned with Ala293 of huCollp289–303. TCR of T cells cross-recognizing huCollp261 and huCollp289–303 will thus possibly contact conserved residues (i.e. Gly265/295, Glu266/296, Gly268/298) all of which are indicated by the above-mentioned study as residues involved in the contact with the TCR. On the contrary, TCRs of T cells selectively recognizing huCollp261 may contact residues that are different between the two peptides, namely Lys264 (replaced by Arg294 in huCollp289-263), whose functional role however appears more relevant in the binding to DR4 than in contacting the TCR [38], and Gln267 (replaced by Hyp297 in huCollp289-263) that is indicated as a main TCR contacting residue in Ref. [38] in agreement with our modeling results.

If the TCR studied here (VB1^OE^) needs Gln267 for recognition of huCollp261, as suggested by our model, peptide huCollp289–303 will fail to stimulate and expand T cells carrying this receptor. This is actually the case.

We cultured PBMC from patient OE in the presence of huCollp261 or huCollp289–303, following the protocol described [15]. Results are shown in panel B of Figure 6, where arrows indicate the peaks corresponding to the product of the VB1^OE^ chain in the immunoscope spectra. T cells carrying the VB1^OE^ chain proliferate in response to huCollp261, and expansion of the corresponding peak (indicated by the arrow) can be observed, as expected. On the contrary, no proliferation is observed when the same cells are stimulated with huCollp289–303. These experimental findings are in line with the results of the computational modeling proposed.

**Figure 7:**
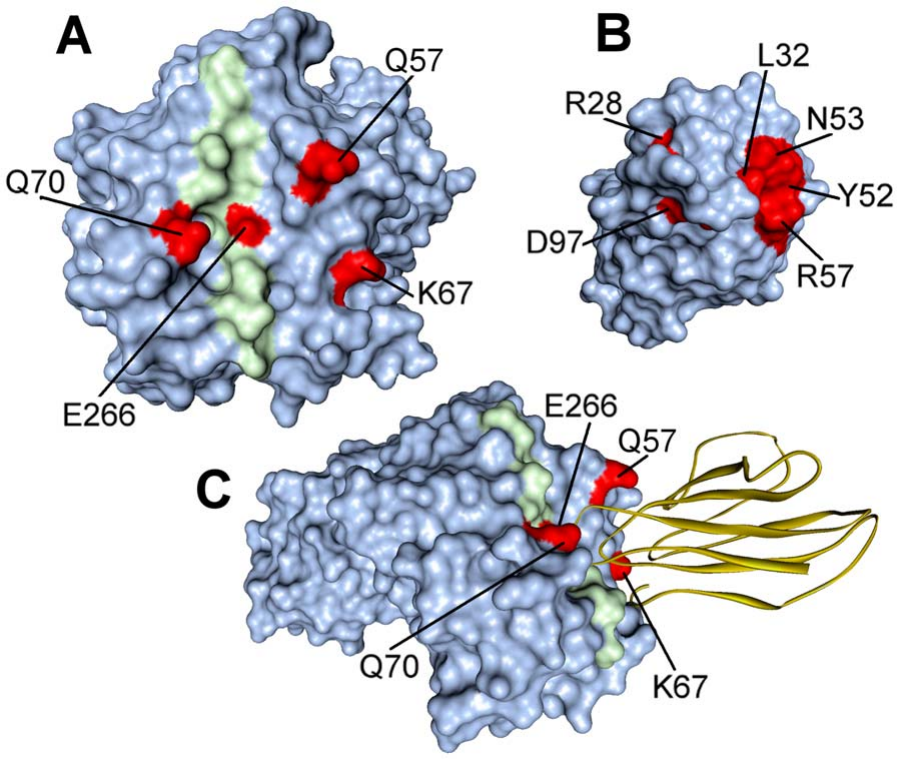
Hotspots predictions in the TCR VB1^OE^ -huCollp261/HLA-DR4 interface. Residues predicted to be hotspots (ΔΔG>1.0 kcal/mol) are shown in *red*, the huCollp261 peptide in *green* and the TCR is represented as a *yellow* ribbon. A) Unbound huCollp261/HLA-DR4 surface; B) Unbound TCR-VB1^OE^ surface; C) TCR-VB1^OE^/huCollp261/HLA-DR4 complex.

The role of individual amino acids in stabilizing the complex was inspected by computational alanine scanning [31]. Residues with ΔΔG>1 kcal/mol are called “hotspots” (Fig. 7) and are listed in Table 6. Recent computational alanine scanning studies [39] have shown that location of the “hotspots” may vary among the various TCR/p/MHC structures. Remarkably, two “hotspots” on TCR VB1^OE^ (Leu32 in CDR1 and Tyr52 in CDR2, see Table 6) interact with DR4α Gln57 and Ala61 (Table 4) which form conserved contacts with TCR Vβ domain [29]. This finding is in close agreement with the alanine scanning analyses of TCR HA1.7 bound to HA/HLA-DR4 and to HA/HLA-DR1 (PDB codes: 1j8h and 1fyt, respectively) [39]. Also computational mutation of CDR3 Asp97 has unfavorable effects on the stability of the complex (Table 6). In the dynamically equilibrated model, Asp97 forms a salt bridge with Arg28 of CDR1 that may be important for maintaining a correctly oriented loop structure (Fig. 5C). We also find that DR4α Gln57Ala is able to cause destabilization at the interface (Table 6, Fig. 5C) and this result is consistent with the above-mentioned structural role of Gln57α. Alanine scanning results also indicate DR4 Gln70β as a residue whose mutation dramatically affects TCR/p/MHC complex. The network of hydrogen bonds established by Gln70β with Ser100, Ser101 and Gly102 of the TCR Vβ domain is shown in Fig. 5B. Taken together, these findings lead to the suggestion that the “shared epitope” region plays an essential role in influencing the strength of T cell recognition. In turn, the strength of the interaction between TCR and p/MHC complex influences polarization of T cells, since a strong stimulation leads to acquisition of the pathogenic Th1 phenotype [40], [41]. Thus the direct engagement of the “shared epitope” by the TCR would contribute to differentiation of T cell specific for huCollp261 towards a pathogenic phenotype and promote the development of RA.

**Table 6:** Computational alanine scanning-based free energies for the TCR-VB1^OE^/huCollp261/HLA-DR4 complex.

Mutation	Protein	ΔΔG_bind_ (kcal/mol)
Arg28Ala	TCR CDR1β	3.92
Leu32Ala	TCR CDR1β	1.33
Tyr52Ala	TCR CDR2β	1.61
Asn53Ala	TCR CDR2β	2.16
Arg57Ala	TCR CDR2β	1.18
Asp97Ala	TCR CDR3β	2.11
Glu266Ala	huCollp261	1.55
Gln57Ala	DR4α	1.96
Lys67Ala	DR4α	1.50
Gln70Ala	DR4β	2.14

### Conclusions

The molecular mechanism of collagenII(261–273)/HLA-DR4 recognition by a TCR Vβ domain characteristic of a DR4^+^ patient affected by rheumatoid arthritis was investigated using molecular modeling, protein-protein docking, and molecular dynamics simulations. It is clear that the possibility of controlling the clonotypic expansion strictly derives from the knowledge of the three-dimensional structure of the complex of TCR with the putative antigen. It is also true that post-transcriptional modifications of collagen can occur that modify the peptide bound to the DR4 molecule. Yet the putative natural antigen unmodified by posttranslational events likely represents the very early initial trigger of the loss of tolerance occurring under genetic control in RA, as observed in the collagen type II induced model of arthritis, possibly along with the posttranslationally modified antigen [42].

Herein, the proposed model finds a correspondence with a large body of existing experimental data and allows the identification of key residues involved in complex stability and specificity. As expected, key residues belong to the region 97–101 of Vβ that distinguishes the TCR of the DR4^+^ patient from that of a DR4^−^ patient. Furthermore, the simulations presented here suggest that the “shared epitope”, common to the RA-predisposing alleles HLA-DR4 and HLA-DR1, directly contributes to the engagement of the TCR itself.

Nowadays, PCR based methods can produce large numbers of sequences of candidate antigen-specific TCR, specially for the β-chain.

The molecular modeling method we describe will prove useful to examine e. g. the variability of the recognition for the same p/MHC complex by different TCRs.

Although the presented strategy should be validated by comparison with mutagenesis experiments involving variations in either peptide or the TCR-Vβ molecule, knowledge of the interactions and key binding residues at the interface between TCR and p/MHC complexes, obtained by pooling information from several of these models, will produce a detailed map of the recognized surface, thereby providing insights into the processes of self- and allo-recognition.

This represents the basis to envision any future strategy to develop tools capable of damping the autoreactivity or to switch-off the autoreactive signal occurring from the interaction.
